# Supplementary material for: Mutational signatures of non‐homologous and polymerase theta‐mediated end‐joining in embryonic stem cells
Source: EMBO J. 2017 Oct 27;36(24):3634–49. doi: 10.15252/embj.201796948 (PMC5730883; doi:10.15252/embj.201796948)
Supplement: Supplementary file 1 — Appendix [file EMBJ-36-3634-s001.pdf]

Appendix

Table of contents

Appendix supplementary legends Page 2

Appendix Figure S1 Page 3

Appendix Figure S2 Page 4

Appendix Table S1 Page 5

Appendix Table S2 Page 6

Supplementary Figure 1. Formation of tandem duplications

A) Model to explain that *de novo* DNA synthesis (indicated in blue) combined with ssDNA resection (strikethrough) results in the formation of a tandem duplication (underlined) upon the induction of a DSB with 5’ ssDNA overhangs by Cas9-D10A (cut sites indicated by red triangles). B) Graphical illustration to explain how a complex arrangement can be the result of iterative rounds of nicking and DSB repair: re-cutting the tandem duplication that results from repair of the initial DSB can result a repair product that will be annotated as deletion (dashed line) with a templated insert (underlined sequence) by our bio-informatical classification software.

Supplementary Figure 2. Validation of Pol λ and Pol μ knockout cell-lines and immunoblots Cas9-nickases *HPRT* assay

A) Immunoblots to confirm loss of Polymerase Lambda (upper panel) and Polymerase Mu (middle panel) protein expression in the used knockout clones. Polymerase Lambda runs on a similar height as a nonspecific band. An immunoblot for Tubulin is included as a loading control (lower panel). B-C) Immunoblots to confirm equal Cas9 protein expression between samples (upper panels), which corresponds with Figure 6B and 6E respectively). Immunoblots for Tubulin are included as a loading control (lower panel).

Table S1. Overview of guide RNAs and oligonucleotides used

Table S2. Absolute *HPRT* mutation frequencies

Appendix Figure S1

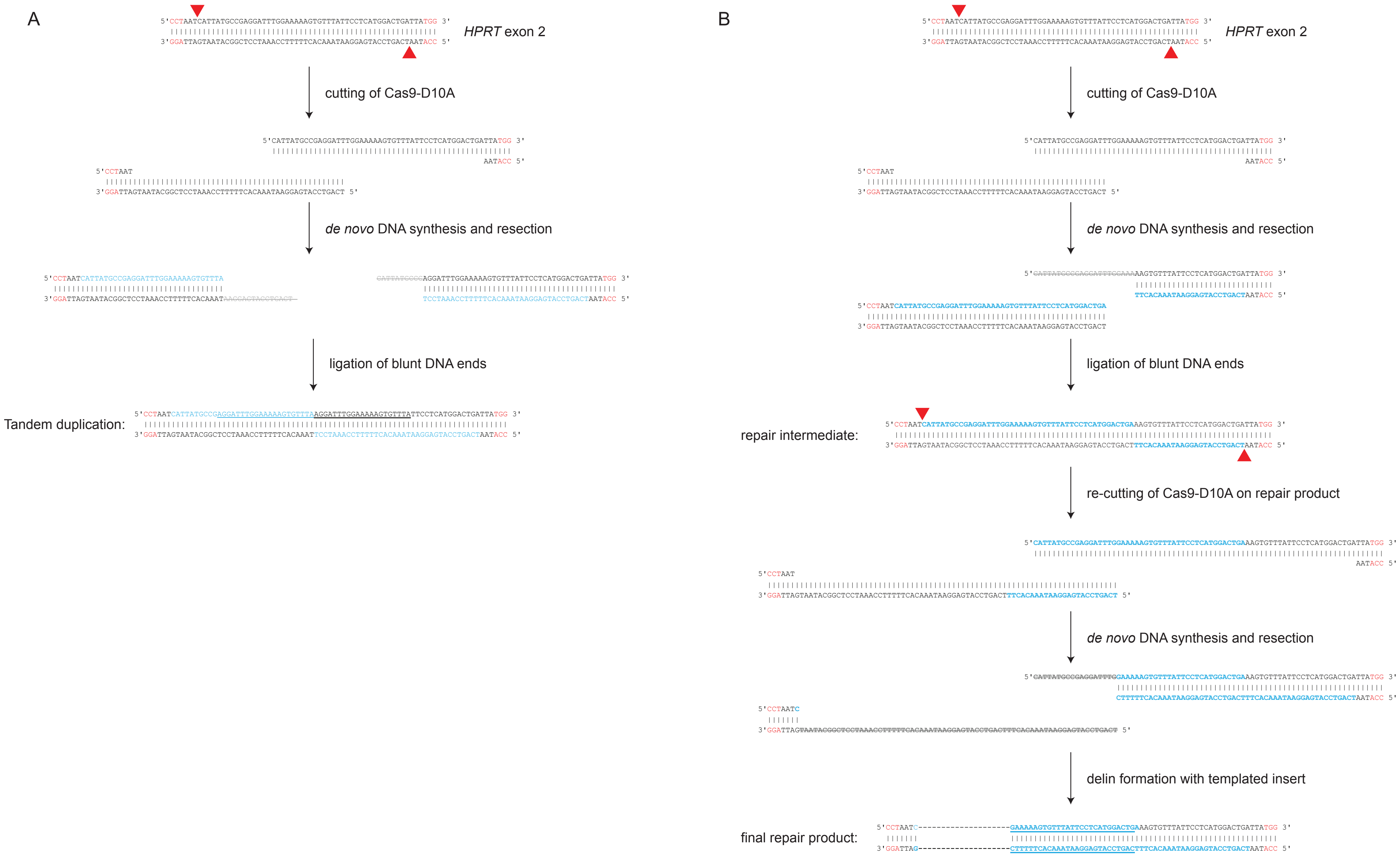

Appendix Figure S2

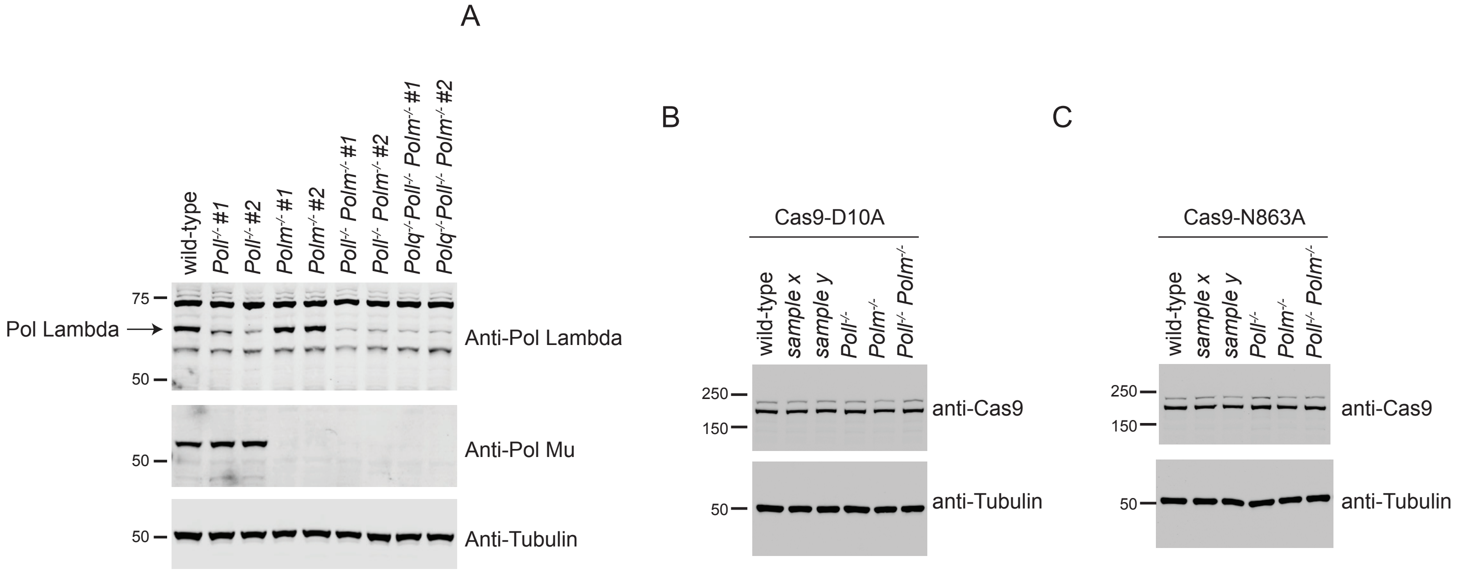

Appendix table S1

Gene	sgRNA	Exon	Forward oligonucleotide for genomic PCR (+ nested)	Reverse oligonucleotide for genomic PCR (+nested)	Oligo for sequencing	Restriction site for RFLP	Remark
Polq	GTCCGGCTCCGACACGTTCT	1	GAGGCCGGAAGCCAGATATCAG	CAGCTGTGCATGCTGGCCAAG		AflIII	
Polq	GTGTAAGAAAGATGTTCGAA	2	GCTAGCATTTTTCTGTATAGG	CAAATGTCCTTGTTTCCCTCC		TaqI	
Ku80	GTGGATGTGGGGGTTGCCAT	2	CTTCACCAGTCACATGATTG	CAAAGGATATAGGGCTCCAG		NcoI	
Ku80	CCTCAGCAGCCCGTTCAGCC	5	CAGTTGTCTCTGTGGTGAGC	CCTCAGCTCAGAACAGTTCC		MwoI	
LigIV	GCACAACGTCACCACAGATC	1	GCCAAGGTGCTTACAGAAAG	CAAACTGGTCGGTATAGTTG		BglII	
LigIV	TACAACTATACCGACCAGTT	1	CAACGTCACCACAGATCTGG	CTAAGGATCTCATACCTCTTC		BsrI	
Poll	AGGAGTCACTCACATTGTGG	2	GAGGTCAAGGCAGGAGAATCAGAA (+GCTACATAGTCAGTTTGAGG)	GACTTGCTGGGCTGTGGTTCATC (+GAGGTCACAGAGAGTTAGGG)		DraIII	
Poll	CTGAGCTTGTGCCTGCAGGA	2	GAGGTCAAGGCAGGAGAATCAGAA (+GCTACATAGTCAGTTTGAGG)	GACTTGCTGGGCTGTGGTTCATC (+GAGGTCACAGAGAGTTAGGG)		PstI	
Polm	GGAAGGGACCTCAGCCAAGG	2	CGATGCTTTCCAAGTACATAC (+GTAACATGCCCAAGTGGCTTG)	ATCCTGAAATGGCAGCGGTC (+GTCTCTATTGCTTTCCCAGG)		BglI	
Polm	GGCTCTGGAGACGCTAGCGG	4	CCTGGGAAAGCAATAGAGAC (+GCTGCCATTTCAGGATGACC)	CACTCCAGGATGCTTTCTGAC (+GCTCTGAACCAAGTCTCCTG)		NheI	
HPRT	TATACCTAATCATTATGCCG	2	GAGTAGAAAGTGTAGAGCCG (+GACTCTATTCAGCAGTAAGAC)	CAGGCACTCACACATACAAG (+CAGCAAGAGACACTGATTC)	GACTCTATTCAGCAGTAAGAC		Cas9 WT
HPRT	ACTTGCTCGAGATGTCATGA	3	CAGCAGGATCTTGTTATGTAG (+CTGGGCTATTTAGAGGGATTC)	GGATAGTGTGAGCAAGTTAG (+CTTAGTTCCCTGGGTTCTAC)	CTGGGCTATTTAGAGGGATTC		Cas9 WT
HPRT	AATCATTATGCCGAGGATTTG	2	GAGTAGAAAGTGTAGAGCCG (+GACTCTATTCAGCAGTAAGAC)	CAGGCACTCACACATACAAG (+CAGCAAGAGACACTGATTC)	GACTCTATTCAGCAGTAAGAC		Site A for Cas9 nickase
HPRT	TATTCCTCATGGACTGATTA	2	GAGTAGAAAGTGTAGAGCCG (+GACTCTATTCAGCAGTAAGAC)	CAGGCACTCACACATACAAG (+CAGCAAGAGACACTGATTC)	GACTCTATTCAGCAGTAAGAC		Site B for Cas9 nickase

Appendix table S2

Cas9 Wild-type *HPRT* exon 2 (Fig 1E)

Genotype:		wild-type	Polq-/- #1	Polq-/- #2	Ku80-/- #1	Ku80-/- #2	Lig4-/- #1	Lig4-/- #2	Polq-/- Ku80-/- #1	Polq-/- Ku80-/- #2
Exp	1	6.59E-02	3.05E-02	2.99E-02	nd	nd	nd	nd	nd	nd
	2	1.49E-02	7.33E-03	4.50E-03	1.66E-02	2.77E-02	1.67E-02	2.80E-02	nd	nd
	3	1.28E-01	nd	nd	1.10E-01	8.39E-02	1.05E-01	8.92E-02	nd	nd
	4	2.15E-02	6.25E-03	6.69E-03	4.29E-02	3.65E-02	2.04E-02	2.92E-02	0.00E+00	0.00E+00
	5	3.16E-01	nd	nd	nd	nd	nd	nd	5.84E-02	3.62E-02
	6	1.07E-01	nd	nd	nd	nd	nd	nd	2.37E-03	2.53E-03

Cas9 Wild-type *HPRT* exon 3 (Fig 1F)

Genotype:		wild-type	Polq-/- #1	Polq-/- #2	Ku80-/- #1	Ku80-/- #2	Lig4-/- #1	Lig4-/- #2	Polq-/- Ku80-/- #1	Polq-/- Ku80-/- #2
Exp	1	6.52E-02	2.16E-02	1.85E-02	nd	nd	nd	nd	nd	nd
	2	2.50E-02	1.11E-02	1.26E-02	2.59E-02	2.84E-02	3.58E-02	4.67E-02	nd	nd
	3	1.32E-01	nd	nd	1.17E-01	9.42E-02	1.25E-01	1.01E-01	nd	nd
	4	2.52E-02	4.81E-03	9.13E-03	4.37E-02	3.00E-02	2.20E-02	2.35E-02	6.25E-03	0.00E+00
	5	4.23E-01	nd	nd	nd	nd	nd	nd	3.07E-02	8.67E-02
	6	9.15E-02	nd	nd	nd	nd	nd	nd	2.33E-03	2.21E-03

Cas9 D10A *HPRT* exon 2 (Fig 3A)

Genotype:		wild-type	Polq-/- #1	Polq-/- #2	Ku80-/- #1	Ku80-/- #2	Lig4-/- #1	Lig4-/- #2	Polq-/- Ku80-/- #1	Polq-/- Ku80-/- #2
Exp	1	3.23E-01	2.63E-01	2.69E-01	2.78E-01	3.01E-01	2.33E-01	2.30E-01	nd	nd
	2	2.59E-01	1.54E-01	1.81E-01	1.91E-01	1.77E-01	1.99E-01	2.02E-01	nd	nd
	3	1.77E-01	1.55E-01	1.42E-01	1.57E-01	1.62E-01	2.05E-01	1.96E-01	5.75E-03	3.08E-03
	4	4.42E-02	nd	nd	nd	nd	nd	nd	3.07E-04	1.75E-04

Cas9 N863A *HPRT* exon 2 (Fig 3B)

Genotype:		wild-type	Polq-/- #1	Polq-/- #2	Ku80-/- #1	Ku80-/- #2	Lig4-/- #1	Lig4-/- #2	Polq-/- Ku80-/- #1	Polq-/- Ku80-/- #2
Exp	1	1.19E-01	8.59E-02	8.74E-02	4.90E-02	4.39E-02	6.75E-02	6.16E-02	nd	nd
	2	5.21E-02	2.45E-02	3.20E-02	1.10E-02	9.87E-03	2.30E-02	2.77E-02	7.37E-04	9.90E-05
	3	1.26E-02	nd	nd	nd	nd	nd	nd	6.90E-04	0.00E+00
	4	6.98E-03	nd	nd	nd	nd	nd	nd	0.00E+00	0.00E+00

Cas9 D10A *HPRT* exon 2 (Fig 6B)

Genotype:		wild-type	Poll-/- #1	Poll-/- #2	Polm-/- #1	Polm-/- #2	Poll-/- Polm-/- #1	Poll-/- Polm-/- #2
Exp	1	2.22E-01	3.56E-01	nd	2.43E-01	nd	1.53E-01	nd
	2	2.60E-01	nd	3.06E-01	nd	2.19E-01	nd	2.18E-01
	3	2.62E-01	2.43E-01	nd	1.70E-01	nd	2.04E-01	nd
	4	2.73E-01	nd	1.42E-01	nd	1.82E-01	nd	1.64E-01

Cas9 N863A *HPRT* exon 2 (Fig 6E)

Genotype:		wild-type	Poll-/- #1	Poll-/- #2	Polm-/- #1	Polm-/- #2	Poll-/- Polm-/- #1	Poll-/- Polm-/- #2
Exp	1	4.36E-02	7.83E-02	nd	6.01E-02	nd	4.72E-02	nd
	2	6.76E-02	nd	7.61E-02	nd	4.38E-02	nd	4.14E-02
	3	6.84E-02	6.71E-02	nd	4.49E-02	nd	3.64E-02	nd
	4	2.29E-02	nd	2.67E-02	nd	2.04E-02	nd	1.93E-02
