# Supplementary material for: Mutational signatures of non‐homologous and polymerase theta‐mediated end‐joining in embryonic stem cells
Source: EMBO J. 2017 Oct 27;36(24):3634–49. doi: 10.15252/embj.201796948 (PMC5730883; doi:10.15252/embj.201796948)
Supplement: Supplementary file 2 — Expanded View Figures PDF [file EMBJ-36-3634-s002.pdf]

Expanded View Figures

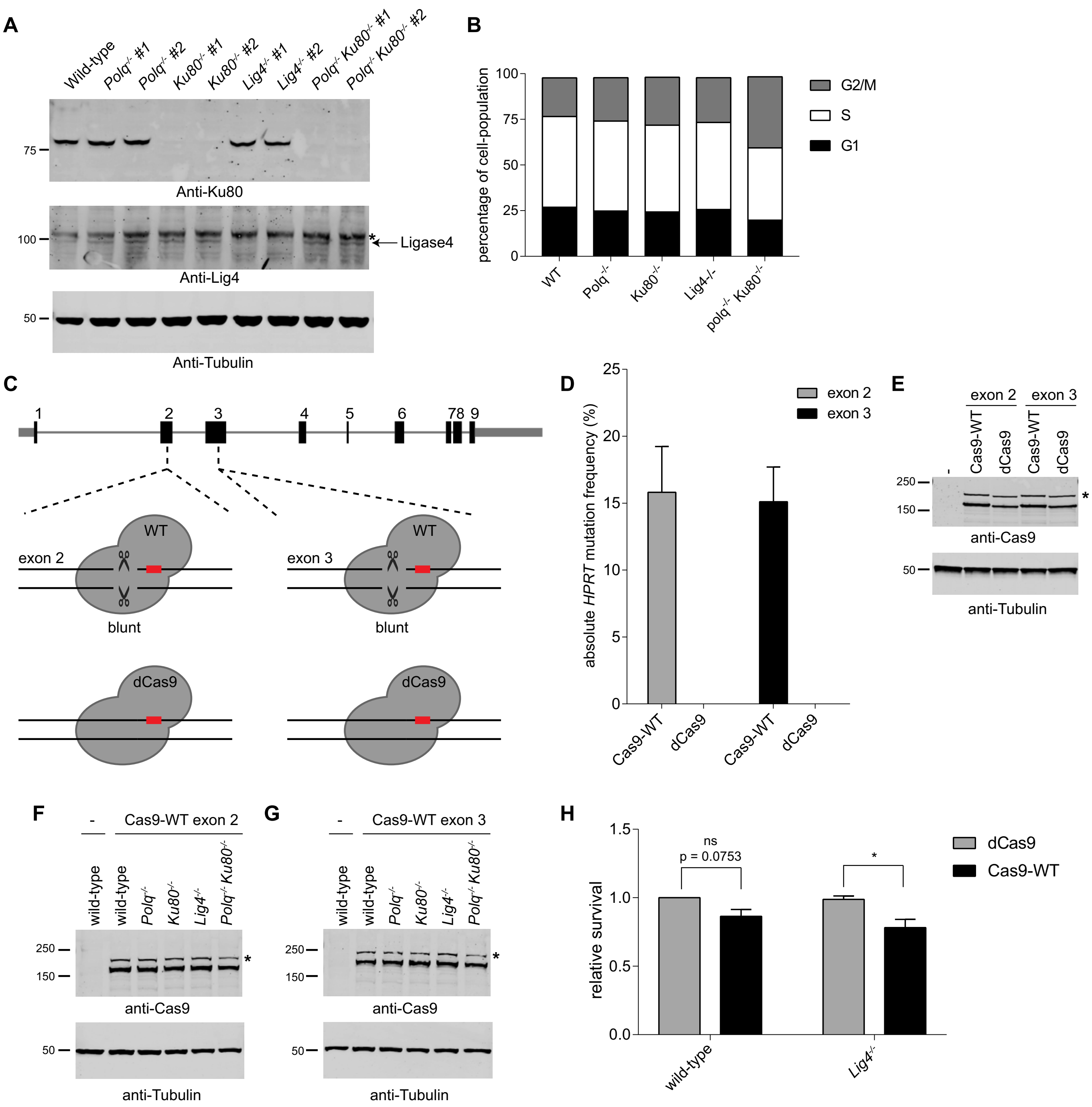

Figure EV1.

Figure EV1. Validation of knockout cell lines and immunoblots for Cas9-WT *HPRT* assay.

A Immunoblots to confirm loss of Ku80 (upper panel) and Lig4 (middle panel) protein expression in knockout clones. An immunoblot for Tubulin is included as a loading control (lower panel). Asterisk on the Lig4 blot indicates a non-specific band.

B Graph showing the cell-cycle phase distribution in the different cell lines for G1, S and G2/M phase as measured by flow cytometry on propidium iodide-stained cells.

C Schematics of Cas9-WT and nuclease-dead Cas9 (dCas9) targeted sequences in *HPRT* exon 2 and exon 3.

D Absolute *HPRT* mutation frequency of wild-type mouse ES cells transfected with Cas9-WT or dCas9 plasmids co-expressing sgRNAs targeting either exon 2 or exon 3 of *HPRT*. Data shown are the mean ± SD ($n = 2$).

E–G Immunoblots to confirm equal Cas9 protein expression between samples (upper panels, corresponding to Figs EV1D, and 1E and F, respectively). Transfection of plasmid px458 in mouse ES cells results in the expression of both uncleaved Cas9-2A-GFP protein (asterisks) as well as cleaved Cas9 protein. Immunoblots for Tubulin are included as a loading control (lower panel).

H Relative cellular survival of wild-type and *Lig4*$^{-/-}$ cells transfected with Cas9-WT compared to the survival of cells transfected with nuclease-dead Cas9 (dCas9). The data shown represent the mean ± SEM ($n = 3$) and are expressed as a fraction of the survival observed in wild-type cells transfected with dCas9 (set to 1). Statistical significance was calculated by two-way ANOVA with Bonferroni correction. ns, not significant, $^*P < 0.05$.

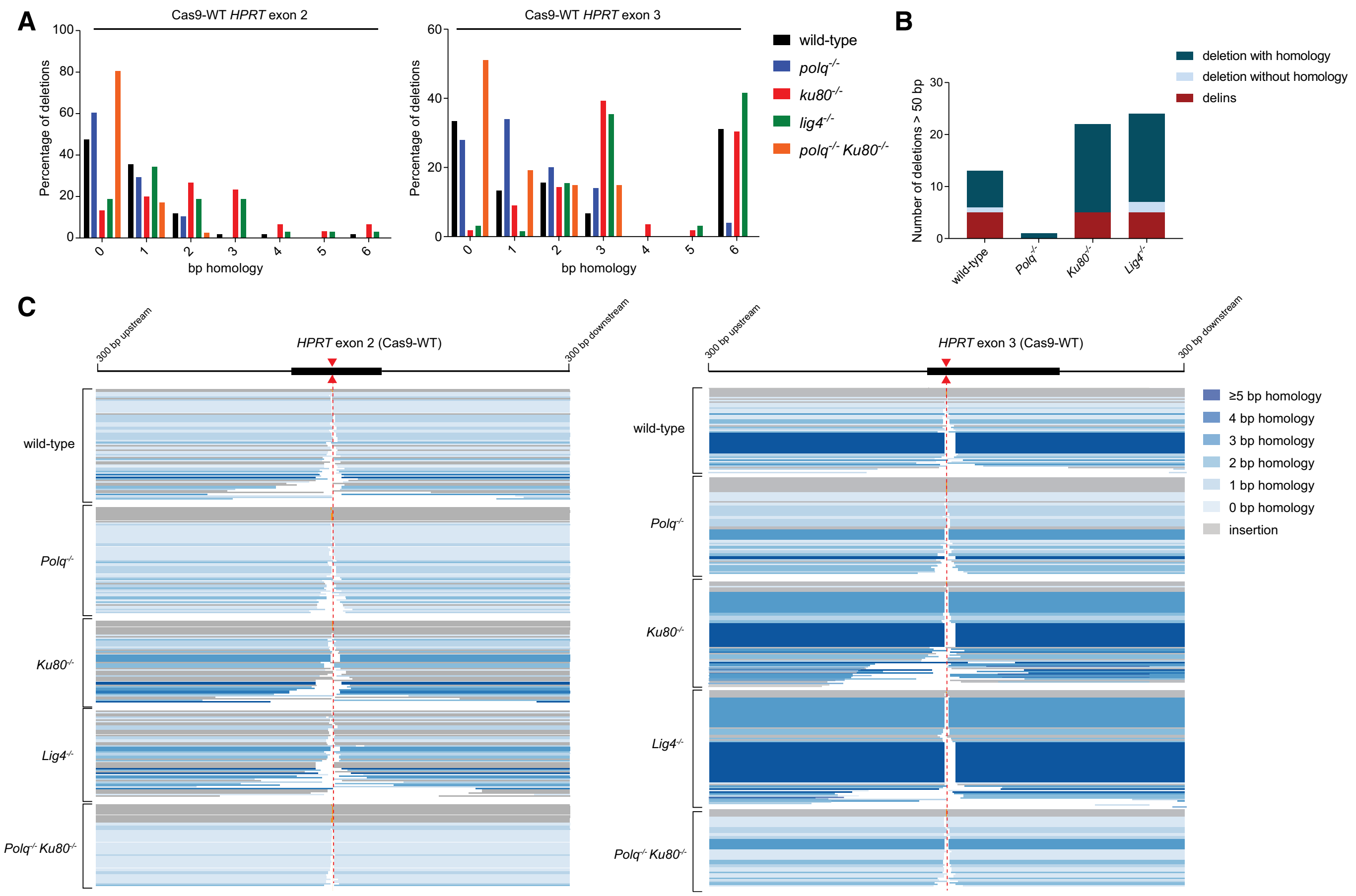

Figure EV2. Extended analysis of Cas9-WT-induced mutations.

A Distribution of homology size (0–6 bp) for the category deletions obtained from different cell lines after the induction of DSBs by Cas9-WT in *HPRT* exon 2 (left panel) or exon 3 (right panel).

B Categories observed in the subgroup of mutations that have a deletions larger than 50 bp.

C Graphical representation of *HPRT* mutations derived from the indicated genotypes. Each bar reflects a single allele obtained from Cas9-WT-induced DSBs in *HPRT* exon 2 (left panel) or exon 3 (right panel) relative to the predicted Cas9 cut site (red dashed line). Simple deletions are colour-coded according to the extent of microhomology used, as indicated in the legend. Grey lines ("insertion") represents both the "delins" as well as the "insertion" category described in Fig 2.

 © 2017 The Authors

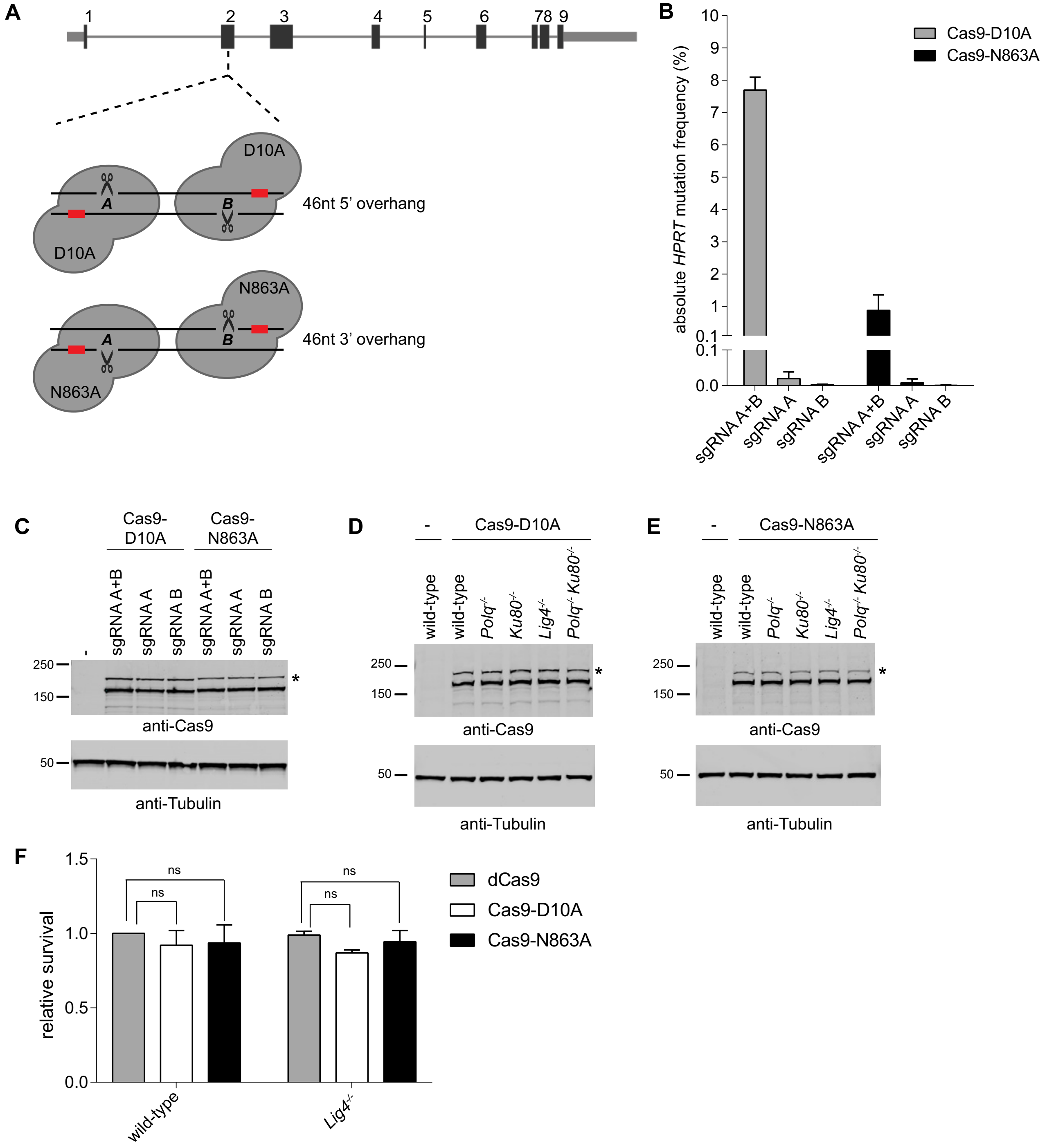

Figure EV3. Validation of the Cas9-nickases *HPRT* assay.

A Schematics of the two Cas9-nickase targeted sequences (A and B) in *HPRT* exon 2 resulting in 46 nucleotide long 5′ ssDNA overhangs (Cas9-D10A) or 46 nucleotide long 3′ ssDNA overhangs (Cas9-N863A).

B Absolute *HPRT* mutation frequency of wild-type mouse ES cells transfected with Cas9-D10A or Cas9-N863A plasmids co-expressing either both sgRNAs (A+B) or only one sgRNA (A or B). Data are the mean ± SD (n = 2).

C–E Immunoblots to confirm equal Cas9 protein expression between samples (upper panels), which corresponds to Figs EV3B, and 3A and B, respectively. Immunoblots for Tubulin are included as a loading control (lower panel). Asterisks indicate uncleaved Cas9-2A-GFP protein.

F Relative cellular survival of wild-type and *Lig4*$^{-/-}$ cells transfected with the Cas9-nickase constructs, compared to the survival of cells transfected with nuclease-dead Cas9 (dCas9). The data shown represent the mean ± SEM (n = 3) and are expressed as a fraction of the survival observed in wild-type cells transfected with dCas9 (set to 1). Statistical significance was calculated by two-way ANOVA with Bonferroni correction. ns, not significant.

© 2017 The Authors

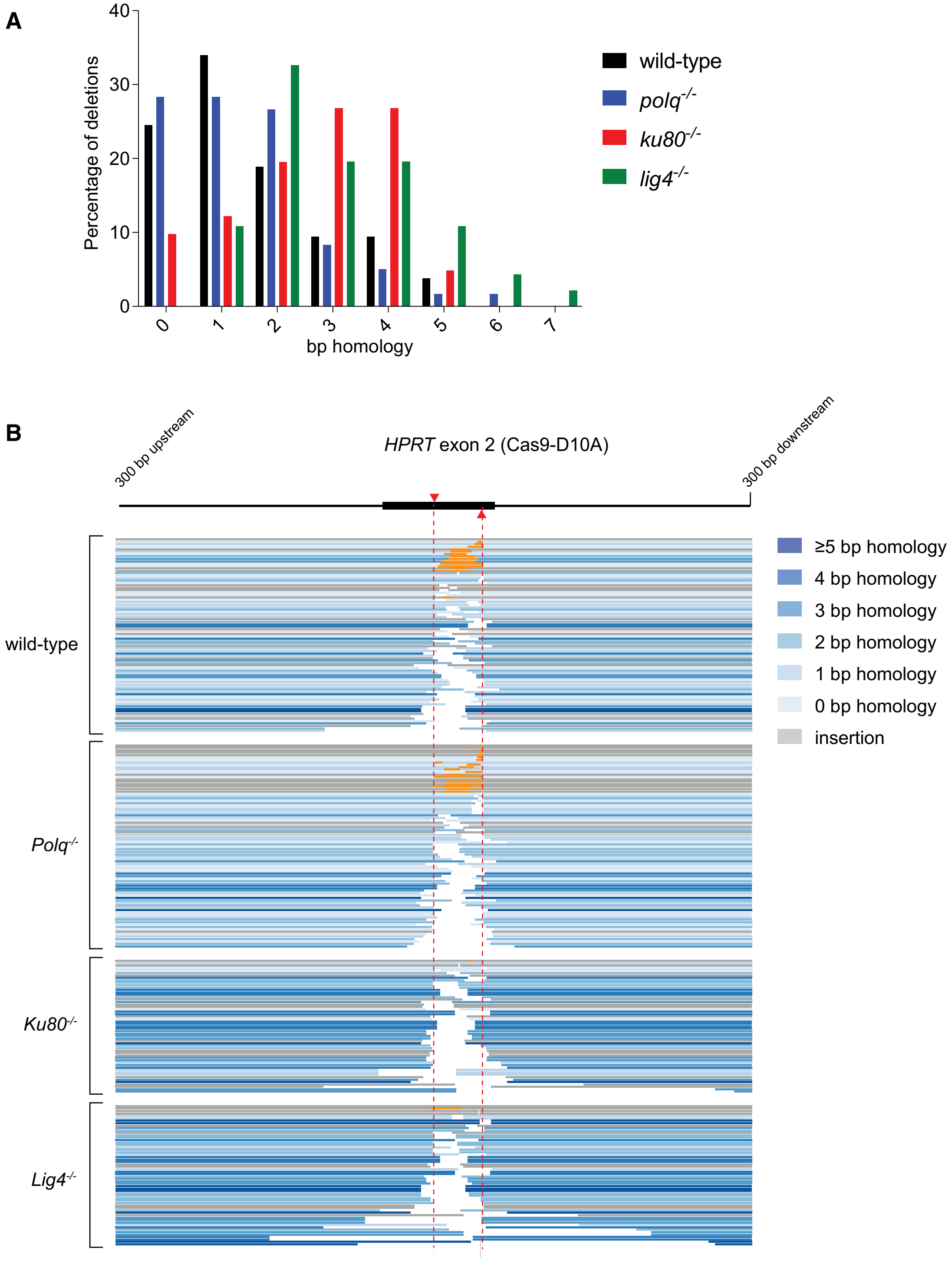

Figure EV4. Extended analysis of Cas9-D10A-induced mutations.

A Distribution of homology size (0–7 bp) for the category deletions obtained from different cell lines after the induction of DSBs by Cas9-D10A in *HPRT* exon 2.

B Graphical representation of *HPRT* mutations derived from the indicated genotypes. Each bar reflects a single allele obtained from Cas9-D10A-induced DSBs in *HPRT* exon 2 relative to the two predicted Cas9 cut sites (red dashed lines). Simple deletions and single tandem duplications are colour-coded according to the extent of microhomology used, as indicated in the legend. All tandem duplications (and other inserts without deletions) are indicated by an overlay between the left flank and the right flank (in orange), where the size of the overlay represents the size of the insert.

 © 2017 The Authors

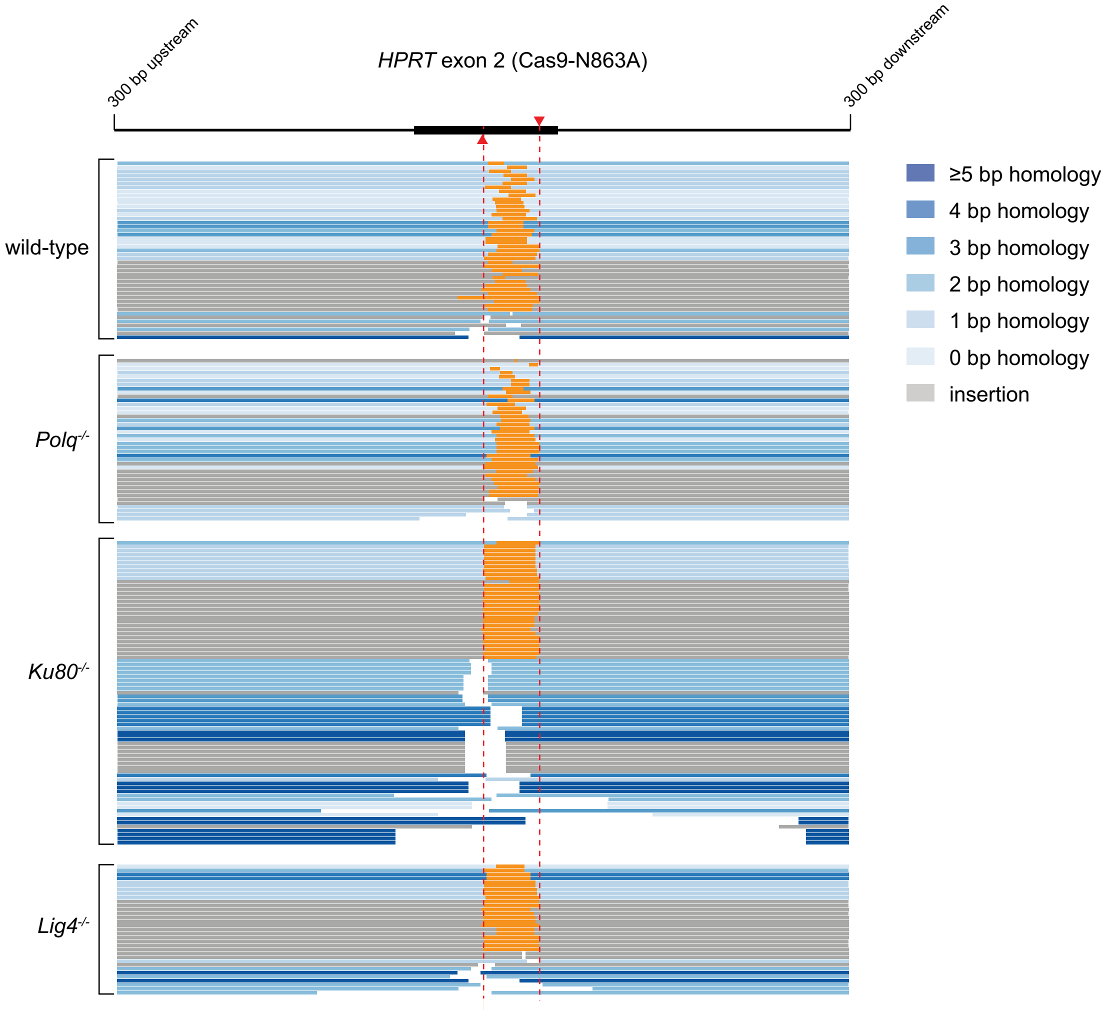

Figure EV5. Extended analysis of Cas9-N863A-induced mutations.

Graphical representation of *HPRT* mutations derived from the indicated genotypes. Each bar reflects a single allele obtained from Cas9-N863A-induced DSBs in *HPRT* exon 2 relative to the two predicted Cas9 cut sites (red dashed lines). Simple deletions and single tandem duplications are colour-coded according to the extent of microhomology used, as indicated in the legend. All tandem duplications (and other inserts without deletions) are indicated by an overlay between the left flank and the right flank (in orange), where the size of the overlay represents the size of the insert.

© 2017 The Authors
